# Direct detection of *Mycobacterium tuberculosis* rifampin resistance in bio-safe stained sputum smears

**DOI:** 10.1371/journal.pone.0189149

**Published:** 2017-12-07

**Authors:** Surabhi Lavania, Divya Anthwal, Manpreet Bhalla, Nagendra Singh, Sagarika Haldar, Jaya Sivaswami Tyagi

**Affiliations:** 1 Department of Biotechnology, All India Institute of Medical Sciences, Ansari Nagar, New Delhi, India; 2 School of Biotechnology, Gautam Buddha University, Yamuna Express way, Greater Noida, Uttar Pradesh, India; 3 Center for Bio-design and Diagnostics, Translational Health Science and Technology Institute, NCR Biotech Science Cluster, 3rd Milestone, Faridabad–Gurgaon Express way, Faridabad, India; 4 Department of Microbiology, National Institute of Tuberculosis and Respiratory Diseases, Mehrauli, New Delhi, India; The University of Hong Kong, CHINA

## Abstract

Direct smear microscopy of sputum forms the mainstay of TB diagnosis in resource-limited settings. Stained sputum smear slides can serve as a ready-made resource to transport sputum for molecular drug susceptibility testing. However, bio-safety is a major concern during transport of sputum/stained slides and for laboratory workers engaged in processing *Mycobacterium tuberculosis* infected sputum specimens. In this study, a bio-safe USP (Universal Sample Processing) concentration-based sputum processing method (Bio-safe method) was assessed on 87 *M*. *tuberculosis* culture positive sputum samples. Samples were processed for Ziehl-Neelsen (ZN) smear, liquid culture and DNA isolation. DNA isolated directly from sputum was subjected to an IS*6110* PCR assay. Both sputum DNA and DNA extracted from bio-safe ZN concentrated smear slides were subjected to *rpoB* PCR and simultaneously assessed by DNA sequencing for determining rifampin (RIF) resistance. All sputum samples were rendered sterile by Bio-safe method. Bio-safe smears exhibited a 5% increment in positivity over direct smear with a 14% increment in smear grade status. All samples were positive for IS*6110* and *rpoB* PCR. Thirty four percent samples were RIF resistant by *rpoB* PCR product sequencing. A 100% concordance (κ value = 1) was obtained between sequencing results derived from bio-safe smear slides and bio-safe sputum. This study demonstrates that Bio-safe method can address safety issues associated with sputum processing, provide an efficient alternative to sample transport in the form of bio-safe stained concentrated smear slides and can also provide information on drug (RIF) resistance by direct DNA sequencing.

## Introduction

Tuberculosis (TB) continues to remain a major global health problem with 10.4 million estimated new TB cases and 1.4 million deaths worldwide in 2015 [1]. Globally, out of the 3,40,000 notified TB patients estimated to have multidrug resistant-TB (MDR-TB), only 40% of them were detected in 2015 [1]. TB diagnosis by direct smear microscopy and culture forms the backbone of TB programmes all over the world. Both techniques suffer from shortcomings; while direct smear has a low sensitivity, culture is time consuming and provides results in a minimum period of 2 weeks [2]. Nucleic Acid Amplification Tests (NAATs) have revolutionized diagnostics by rapidly diagnosing TB with acceptable sensitivity and specificity. There are two categories of commercial NAATs in use, one which detects TB and the other which also provide information on drug resistance status. TB detection is provided by various commercially available NAATs, being used globally, in spite of not being endorsed by the WHO [2]. Recently, Loop-mediated Isothermal Amplification (TB-LAMP) test, has been recommended by the WHO, which detects TB in sputum samples in ~1 hour and has been proposed as a replacement test to sputum smear microscopy [3]. Tests providing information on drug resistance status include the WHO-endorsed Xpert^®^ MTB/RIF (Xpert) and various Line Probe Assays (LPA) [2,4,5]. Xpert (and a more advanced version i.e. Xpert Ultra) is a diagnostic test for TB and Rifampin (RIF) resistance that can be used with minimal technical expertise and provides results within 2 hours [2,6]. However, Xpert suffers from the limitations of high cost, requirement for maintenance, stable electric supply and ambient temperature control, making it unsuitable for resource limited settings and less developed geographical locations [7]. A battery operated version of Xpert i.e. GeneXpert Omni might help in overcoming these limitations in resource limited settings, however this version is still in evaluation phase [2]. On the other hand, although LPA provides information on most MDR and extremely drug resistant (XDR) TB markers, its high sensitivity is impaired by its diligent processing steps and open hybridization format which can cause amplicon contamination [2].

New and novel NAATs cannot undermine the traditional microscopy and culture methods and the molecular assays are usually applied in parallel with these tests for TB diagnosis or drug susceptibility testing (DST). However, performing such molecular tests requires sputum sample storage and safe transportation from remote health centers to Central laboratories (NRLs/IRLs) which is a major challenge in bio-safety or alternatively, movement of sick patients to the Central laboratories to provide a sample. Since a functional network of Designated Microscopy Centers (DMCs) is established under the RNTCP for performing direct smear microscopy, the DMCs offer ready-made sites for processing sputum for molecular tests. Studies have shown the utility of NAAT-based TB detection and/or drug resistance status directly from Ziehl-Neelsen (ZN) or auramine-stained slides; but none of these studies incorporate bio-safe processing of samples [8–12]. Moreover, in resource-limited settings, where access to BSL-3 level laboratories is limited, the safety of laboratory personnel is a major concern. Although some studies have demonstrated methods for the inactivation of the sputum smear [13,14], no study to the best of our knowledge, has attempted to develop a bio-safe sputum processing method and its compatibility with DNA extraction and drug resistance testing.

A bio-safe sample processing method was previously developed and evaluated for smear microscopy at the All India Institute of Medical Sciences, New Delhi (AIIMS) [15]. In the present study, we employ this method to demonstrate the workflow from Bio-safe sample processing to successful isolation of *Mycobacterium tuberculosis* (*M*. *tuberculosis*) DNA and determination of rifampin resistance directly from sputum as well as from stained slide smears. The aims of this study were (i) bio-safe processing of sputum samples for smear and DNA isolation and (ii) assessment of rifampin resistance using DNA isolated directly from sputum samples and smear slides after bio-safe processing.

## Materials and methods

### Study design and sample processing

**Fig 1:**
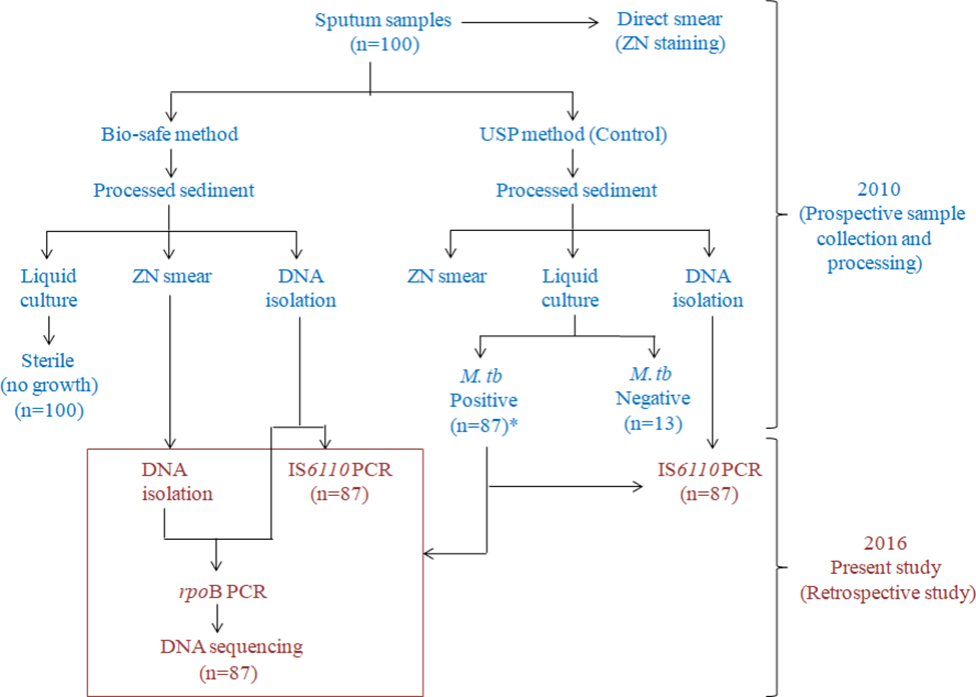
Workflow of the study showing timeline for sample collection and processing in this study. *Only *M*. *tuberculosis* culture positive samples were retrospectively selected for the current study.

The current study was done retrospectively on archived DNA samples and stored bio-safe smear slides from 87 *M*. *tuberculosis* culture positive sputum specimens. These samples (n = 87) were a subset of 100 specimens collected prospectively during October 2010 to December 2010 at the National Institute for Tuberculosis and Respiratory Diseases (NITRD), without any prior knowledge of their treatment status. A timeline of sample collection and processing has been included as Fig 1. Briefly, a direct smear of each sample was prepared for ZN staining prior to sample processing. Then, all samples were processed by a bio-safe Universal Sample Processing (USP) concentration-based sputum processing method (Bio-safe method) [15] for ZN smear, culture and DNA isolation. In parallel, the USP method was used as a control [16] **(Fig 1**). Briefly, USP solution (2.5 volumes of 6M guanidine hydrochloride, 25 mM EDTA, 0.5% Sarkosyl, 50 mM Tris-Cl, pH 7.5 and 0.1 M β-mercaptoethanol) was added to each sputum sample (~4ml), and vortexed as described [16]. The liquefied sputum was divided into two parts; 5% phenol (final concentration) was added to one part (Bio-safe method) and other part was used as control (USP method). Both aliquots were incubated at room temperature for 30 min, after which 25–30 ml sterile water was added and mixed. The sample was centrifuged for 15 min and the sediment was washed with USP solution and re-centrifuged. In the Bio-safe method, the sediment was additionally given an ethanol wash and finally both pellets were given a water wash. The processed sediment was used for ZN smear microscopy, culture, and DNA isolation (**Fig 1**). These bio-safe smear slides and sample DNA from these specimens were stored in the AIIMS laboratory **(Fig 1)**.

### Smear microscopy, *M*. *tuberculosis* culture and DNA isolation

For direct smear microscopy, a loopful (5 mm diameter) of sputum sample was taken for smear preparation in an area of 2 by 3 cm, then air dried and heat fixed. The processed sediments were re-suspended in 300 μl of resuspension solution (0.05% Tween-80) of which 10% was used for smear preparation. All smear slides were stained with ZN stain, observed and graded as per the Revised National Tuberculosis Control Programme (RNTCP) guidelines [17]. For culture, 45% of the suspension (135 μl) was inoculated into 7H9 liquid medium supplemented with Albumin Dextrose complex and PANTA (polymyxin B, amphotericin B, nalidixic acid, trimethoprim and azlocillin) supplement (Becton Dickinson, USA), and the tubes were incubated at 37°C for upto 8 weeks. The positivity in the 7H9 liquid culture was first identified on the basis of turbidity of the medium, which was then confirmed for presence of *M*. *tuberculosis* by ZN smear microscopy and IS*6110* PCR (*M*. *tuberculosis* complex specific PCR) [18]. For DNA isolation, the remaining 45% of the suspension (135 μl) was pelleted and re-suspended in 250 μl lysis solution (10% Chelex-100, 0.03% Triton X-100 and 0.3% Tween-20), heated at 90°C for 40 min., and centrifuged at ~8800×*g* for 10 min. The supernatant was used directly for PCR.

### DNA isolation from smear slides

DNA was also extracted directly from all bio-safe smear slides (**Fig 1**). Briefly, mineral oil was removed with xylene, and the smears were scraped off the slides using 25 μl of sterile distilled water and transferred to 1.5 ml micro centrifuge tubes. Seventy-five μl of lysis solution (15% Chelex-100, 0.03% Triton X-100 and 0.3% Tween-20) was added, thoroughly mixed and the suspension was heated at 90°C for 40 min. The samples were then centrifuged at ~8800×*g* for 10 minutes. DNA in the supernatant was purified and concentrated by using DNA Clean and Concentrator^TM^-5 kit (ZymoResearch, Irvine, USA). DNA was eluted in 20μl of elution buffer and used directly for PCR and DNA sequencing.

### IS*6110* PCR

IS*6110-*specific PCR was performed with DNA obtained by both Bio-safe method and USP method (Control arm). Briefly, 10 μl of specimen DNA was amplified in a 40 μl reaction containing 0.5 μM of each of the primers T4 and T5 [18], 0.2 mM dNTPs, 1× PCR buffer, 1.5 mM MgCl_2_, 1 U of Taq DNA polymerase (iNtRON; Korea). The thermal cycling parameters were 10 min at 94°C, 45 cycles of 1 min at 94°C, 30 seconds at 60°C, and a final extension of 7 min at 72°C. The PCR product (123 bp) was checked by agarose gel electrophoresis. PCR inhibition was assessed for each sample by adding 20 to 30 ng of pure *M*. *tuberculosis* H37Rv DNA into an additional reaction containing specimen DNA.

### Detection of drug resistance: Amplification and sequencing of *rpoB* gene

DNA obtained after bio-safe processing from both processed sediment and ZN smear slide was used as template for sequencing. The 261-bp region encompassing the 81-bp rifampin resistance determining region (RRDR) of *rpoB* gene was amplified using primer pairs as previously described [19]. The PCR parameters were 5 min at 94°C, 40 cycles of 30 seconds at 94°C, 30 seconds at 62°C, 30 seconds at 72°C, and a final extension of 7 min at 72°C. Amplified PCR products from both bio-safe sample DNA (all 87 samples) and slide DNA (20/87 samples) were purified by using PCR Clean-up kit (Promega) and sequenced. For sequencing, a 10 μl reaction mixture containing BigDye Terminator chemistry v3.1 kit (Applied Biosystems, USA), 4 pM of *rpoB* seq primer, and 30–40 ng of cleaned PCR product was used. The thermal cycling parameters were 25 cycles of 10 seconds at 96°C, 5 seconds at 50°C and 4 minutes at 60°C. The amplified products were further purified using the ethanol precipitation method and then sequenced in 3130xl automated DNA analyser (Applied Biosystems, Inc., Foster City, California, USA).

### Statistical analysis

The various tests employed in the study were compared on the basis of their sensitivity, specificity and concordance. IS*6110* PCR results were assessed using culture as a reference standard. IS*6110* PCR sensitivity was defined as [True positives] / [True positives + False negatives] ×100; wherein true positives were defined as samples positive by both IS*6110* PCR and culture, and false negatives were culture positive samples missed by IS*6110* PCR. Specificity was defined as [True negatives] / [True negatives + false positives] ×100; wherein true negatives were defined as samples negative by both IS*6110* PCR and culture, and false positives were samples positive by IS*6110* PCR but negative by culture. Concordance analysis was performed to assess the near-equivalence of Bio-safe method and USP (control) method and also between direct Bio-safe sputum sequencing and ZN smear slide sequencing [20]. Concordance was calculated as [True positives + true negatives] / [total number of samples] ×100 [20]. The degree of concordance/agreement was measured by Cohen’s kappa (κ), which was calculated as described [21]. The various mutations detected by sequencing in both sputum samples and slides DNA were plotted using GraphPad Prism version 6.00 for Windows, (GraphPad Software, La Jolla California USA, www.graphpad.com).

### Ethical statement

All samples were collected after obtaining ethical clearance from the Institutional Ethics Committees of All India Institute of Medical Sciences and National Institute of Tuberculosis and Respiratory Diseases hospitals. These sputum samples were collected after written informed consent was taken from patients. All the patients included in this study were adults.

## Results

### Smear microscopy and culture

**Fig 2:**
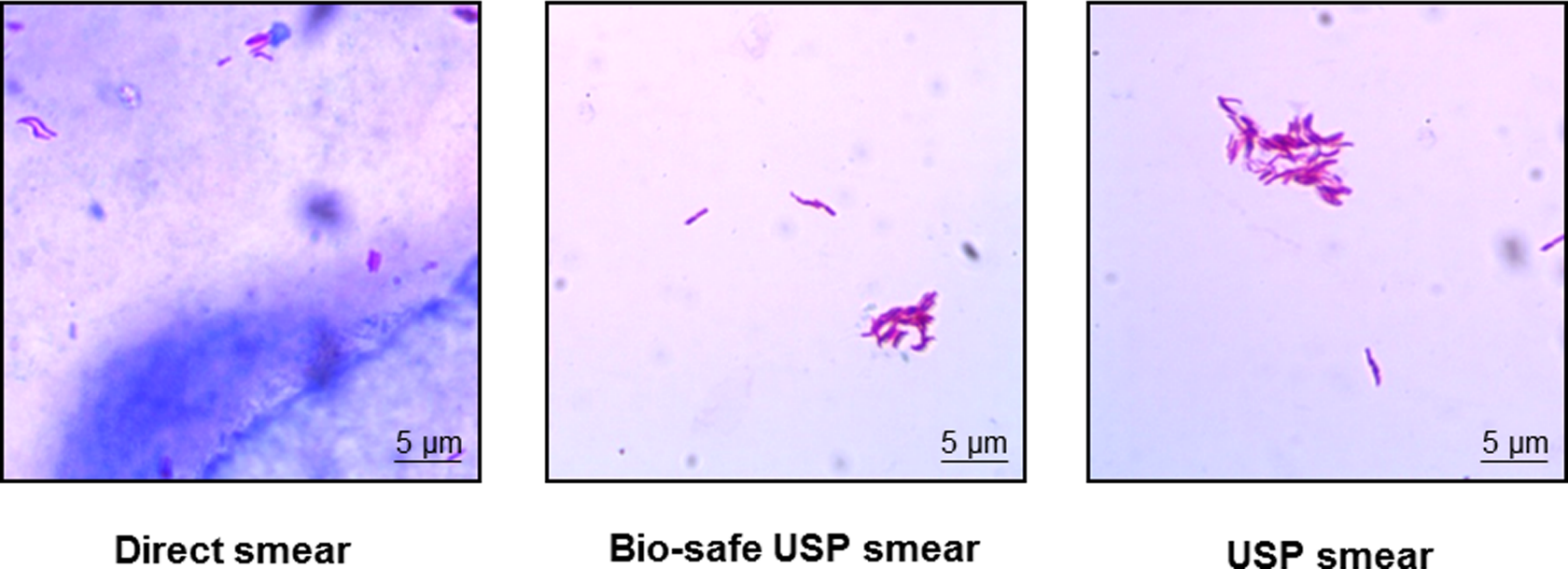
Direct, USP and bio-safe USP smear. USP and bio-safe USP smears show a clear background as compared to direct smear.

Out of 87 samples, 83 were direct smear positive. A 100% positivity was obtained by Bio-safe USP method and the step of concentration of sputum improved sample positivity by ~5% as compared to direct ZN smear. The increase in positivity was also accompanied by an increase in smear grade status and clear slide background in comparison to direct smear (**Fig 2**). An increment of 14% was found from grade status of 1+ in direct smear to 2+/3+ by bio-safe smear (**Table 1**). The USP method (control arm) gave identical results, indicating no adverse effect of bio-safe processing on sample positivity and grade enhancement (**Table 1**). All 87 samples were sterile in liquid culture by the bio-safe USP method (confirming bio-safety) and culture-positive by the corresponding USP control.

**Table 1:** Smear status of culture positive sputum samples.

Grade	Bio-safe smear	USP smear	Direct smear
**3+**	30	27	17
**2+**	20	23	20
**1+**	37	37	46
**Negative**	0	0	4
**Any positive**	87	87	83

### PCR assays

DNA isolated from the Bio-safe and USP methods were subjected to IS*6110* PCR and 100% sensitivity was obtained by both methods. For molecular drug susceptibility testing (DST), *rpoB* PCR was performed using DNA isolated from bio-safe smear slides and processed sputum (**Fig 1**). All the samples were positive by *rpoB* PCR and amplified DNA was sequenced for determining RIF resistance/susceptibility. We could not estimate the specificity of these PCR assays (IS*6110* and *rpoB*) due to the lack of *M*. *tuberculosis* culture-negative samples in the study. However, we wish to reiterate that the primers utilized for IS*6110* and *rpoB* PCR assays are specific for the *M*. *tuberculosis* complex and do not amplify DNA of any other mycobacterial species. Also, the specificities of these assays have been established previously in published studies [18,19].

### Direct detection of rifampin resistance by DNA sequencing

**Fig 3:**
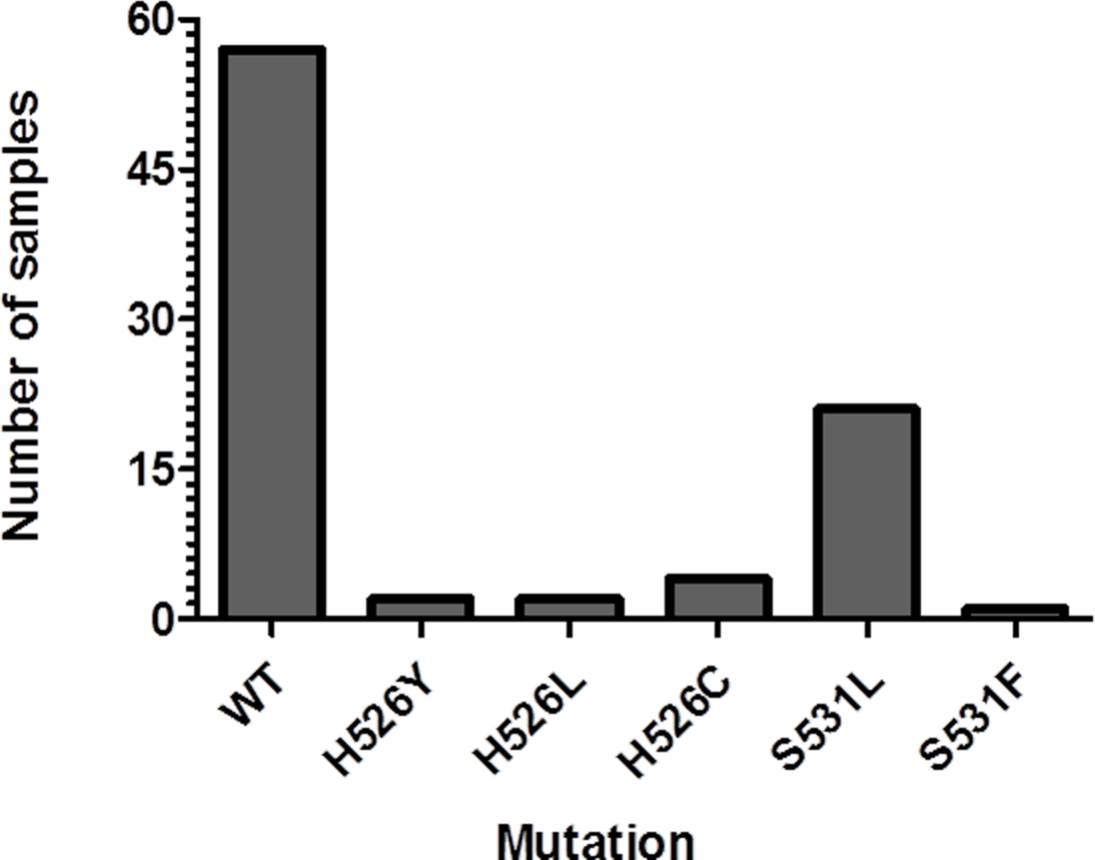
Direct detection of *rpoB* mutations in bio-safe sputum samples (n = 87) by DNA sequencing.

Out of 87 samples, 57 samples were WT in the *rpoB* RRDR region and 30 samples showed mutations at the 526 and 531 codons. Twenty two samples had a mutation at the 531 codon (TCG→TTG [Ser→Leu; 21 samples] and TCG→TTC [Ser→Phe; 1 sample]) and eight samples carried an altered codon 526 (CAC→TGC [His→Cys; 4 samples], CAC→TAC [His→Tyr; 2 samples] and CAC→CTC [His→Leu; 2 samples] (**Fig 3**). The sequencing results of bio-safe slide DNA were 100% concordant (κ = 1) with that of bio-safe sputum DNA.

## Discussion

In resource limited countries like India, sputum smear microscopy is used as the primary tool for TB diagnosis as it is rapid, simple and cost-effective. While sputum needs ambient temperature for transportation, smear microscopy slides can be stored and transported at room temperature for further tests such as PCR and molecular DST [8]. However, this poses a serious bio-safety hazard for laboratory workers [22]. Viability testing from a previous study has shown that 5% Phenol was effective only when the sample was incubated with the disinfectant for 30 minutes [15]. But in normal practice, the sputum smear on the glass slide is kept in contact with ZN stain containing 5% phenol for 5 to 7 minutes only. Also, an earlier study has shown that heat-fixed stained sputum smears contains viable bacilli [22]. On the contrary, our Bio-safe USP method completely disinfects the sputum smears, thereby allowing transportation of stained smears with improved safety. Here, we propose that a bio-safe smear slide that is prepared for smear analysis can then be directly transported after viewing for molecular drug susceptibility testing (MDR/XDR testing). This augmented bio-safe processing step has the added advantage of providing the patient with the DST results within a few days rather than a few weeks to months by conventional liquid culture method. The Bio-safe sample processing method used in the present study has a four-fold advantage; namely provides safety to laboratory workers performing smear microscopy, increases the sensitivity of smear microscopy by concentration, bio-safe transportation of sputum/smear slides to a higher level laboratory (NRL/IRL) and is compatible with DNA isolation and sequencing for molecular DST tests.

All 87 samples were positive by bio-safe smear microscopy method and showed a 5% increase in positivity over direct smear microscopy. RNTCP utilizes direct smear microscopy for case detection and it is considered that a 5% increment in detection sensitivity will enhance case detection and treatment and thereby have an impact on disease transmission [23]. An increase in positivity of concentrated smear microscopy has been reported earlier [23] but has not been implemented for routine diagnosis. The increment in positivity of concentrated smear microscopy and in smear grade status (**Table 1**) along with a clean slide background observed in the bio-safe/ USP smear (**Fig 2**) facilitates slide observation by the technician and improves productivity of reporting the results.

The Bio-safe method which is a modified version of a previously published protocol for sputum processing [16] and utilizes phenol, an effective disinfectant for mycobacteria [13–15,24] and is used for laboratory disinfection and safe disposal of TB-infected material under the RNTCP [17]. Initial viable bacterial count and subsequent loss of viability after the disinfection step (bio-safe USP method) has already been established. It was previously shown that as compared to USP solution, none of the corresponding aliquots having initial bacterial loads of 1 x 10^4^ to 6 x 10^7^ CFU/ml and were treated with USP solution+phenol (bio-safe USP method), yielded any growth of AFB in liquid culture. It was also shown that phenol-mediated disinfection did not compromise smear microscopy [15]. Therefore, it is proposed that smear prepared by the bio-safe method will provide bio-safety to laboratory personnel performing smear microscopy and also provide improved bio-safety during the transportation of sputum/smear slides. Notably, Bio-safe method of sputum processing was also compatible with DNA isolation, PCR amplification and sequencing.

In the present study, the most common mutations were at the codon 531 (S531L, 21 samples and S531F, 1 sample) and codon 526 (H526C, 4 samples; H526Y, 2 samples and H526L, 2 samples) (**Fig 3**). All these mutations are located in the RRDR of *rpoB* gene, which accounts for ~95% of mutations causing RIF resistance [25]. These mutations have been reported previously at a frequency of 47–81% (531 codon), 7–45% (526 codon), and 5.6 to 22% (codon 516) in various Indian studies [26–31] and contribute to 70–95% of total *rpoB* gene mutations leading to RIF resistance [25,31]. However, in our study the two codons (526 and 531) alone contributed to 100% of RIF resistance. The possible reason for this result may be the small sample size in our study. The reason for the high numbers of RIF resistant samples in our study (~34%) can be explained firstly, by the fact that anti-tubercular therapy (ATT) status of these samples was not known prior to analysis, and secondly, the samples used in this study were collected from NITRD hospital, which is a tertiary care hospital, and several of the patients attending the Hospital are previously treated [32]. High levels of resistance have been reported previously from tertiary care hospitals in both new and previously treated TB cases [33,34].

Sequencing results of bio-safe slide DNA were in complete concordance with results directly from bio-safe sample DNA established the compatibility of slide DNA with DNA sequencing. Extracted and purified DNA was stable and of good quality, enabling the performance of molecular tests, such as PCR and sequencing for detecting TB and drug resistance. Previously, other studies have also explored the possibility of TB detection and drug resistance determination directly from smear slides by PCR, LPA, MAS-PCR and DNA sequencing [8–10,12], but none of them have employed bio-safe sample processing.

The major strength of this study is that, a concentrated bio-safe smear microscopy method compatible with providing drug resistance information was optimized. This approach ensures safety of laboratory personnel. The adoption of bio-safety practices is an unmet need in view of reported incidence of tuberculosis among laboratory workers handling clinical specimens [35]. A smear microscopy slide can potentially serve as a ready source of DNA, unlike a conventional method such as culture which takes several weeks to provide results. The WHO update on MDR-TB, 2015 [36] cites rapid testing and detection of drug resistant TB as a priority action. The method described in the present study addresses this challenge, bypasses delays due to culturing and enables rapid diagnosis of RIF resistant TB from stained sputum smears. Importantly, the method can be extended to the rapid diagnosis of resistance to other TB drugs including isoniazid, fluoroquinolones and aminoglycosides.

The limitations of this study were the non-availability of prior ATT status of the patients included in this study and phenotypic DST results to confirm rifampin resistance data obtained by sequencing. In addition, we cannot comment on whether the bio-safe component will be compatible with the most common decontamination method used for sputum processing i.e. the NALC-NaOH method. NALC-NaOH method and bio-safe USP method are two independent alternative methods for sputum processing. The bio-safe USP method is a modification of the USP methodology developed previously in the AIIMS laboratory [16]. The USP methodology has been previously compared with NALC-NaOH method in our laboratory and both the methods were found to be equivalent in their decontamination efficacy [16]. Hence, this comparison was not made in the present study. Moreover, since NALC-NaOH method and USP method employ different reagents, we have not investigated whether the bio-safe component will be compatible with NALC-NaOH method. Another drawback of this study was that these samples were collected from a National Reference Laboratory (National Institute of Tuberculosis and Respiratory Diseases, New Delhi) where most of the patients attending the hospital are referred presumptive cases of TB, so sample bias cannot be ruled out.

## Conclusions

The Bio-safe sputum processing method described here is expected to find use in settings where non-infectious stained smears prepared in diagnostic laboratories including Designated Microscopy Centers (DMCs) can be safely transported without specialized container requirements to a Central laboratory for molecular testing. The adoption of this two-tier approach is expected to facilitate improved TB detection and determination of rifampin drug resistance directly from sputum smears, with the feasibility to extend drug resistance reporting for other anti-TB drugs. Thus coupling of smear microscopy diagnosis at the peripheral-level laboratory and implementation of molecular tests at the NRL/IRL will hasten the generation of DST data without the requirement for patient travel and any specific upgrade in infrastructure or training at the DMCs. Molecular methods such as Xpert MTB/RIF assay, TB-LAMP and Line Probe Assays (LPA) are emerging as the first diagnostic test to be performed on sputum [2–5]. Genotypic methods like sequencing are also poised to replace phenotypic methods in the future [37]. With the rapid development of sequencing tools and a decrease in their cost, a Bio-safe sample transportation system combined with centralized molecular DST has the potential to provide customized treatment to patients, including in remote areas. Since genotypic methods are quick and provide ‘sample to answer’ in a few hours to days we believe the bio-safe method has a utility in a clinical setting especially because no separate sample is required for DNA isolation and the DST can be done from ZN stained slide itself. Further studies are required to evaluate its utility in low- and middle-income countries for national TB programmes.
